# Supplementary material for: ActiTect: a generalizable machine learning pipeline for REM sleep behavior disorder screening through standardized actigraphy
Source: NPJ Digit Med. 2026 Jun 13;9:647. doi: 10.1038/s41746-026-02738-8 (PMC13498587; doi:10.1038/s41746-026-02738-8)
Supplement: Supplementary file 1 [file 41746_2026_2738_MOESM1_ESM.pdf]

Supplementary Data of *"ActiTect: A Generalizable Machine Learning Pipeline for REM Sleep Behavior Disorder Screening through Standardized Actigraphy"*

Contents

- **A:** Cross-Cohort Generalization of Existing Methods (p. 1)
- **B:** List of Engineered RBD Motor Pattern Features (p. 3)
- **C:** Hyperparameter Search Space (p. 4)
- **D:** Per Dataset Ranking Correlation (p. 4)
- **E:** Multi-Center Model Validation (including Local Test) (p. 5)
- **F:** Statistical Significances of Presented Features Per Dataset (p. 6)
- **G:** Medication Prevalence Per Dataset (p. 7)

Supplementary A Cross-Cohort Generalization of Existing Methods

To assess the transferability of existing actigraphy-based RBD classifiers, we began by evaluating RBDAct, a previously published machine learning model developed to detect RBD in patients with PD using wrist actigraphy [1]. The method demonstrated high classification performance in internal cross-validation, distinguishing patients with PD+RBD from those with Parkinson's disease without RBD and healthy controls. However, whether RBDAct maintains its performance across independent cohorts has not yet been tested; in this study, we perform an external-validation analysis to quantify its cross-cohort generalizability.

Leveraging the code provided by the original authors, we deployed all 100 off-the-shelf models—each pretrained on the source cohort during their internal cross-validation—directly on our independent datasets. Further, we re-trained the RBDAct pipeline on our iRBD versus healthy-controls recordings using the authors' codebase. These models are referred to as iRBDAct in the following.

For our experiments we had to apply the following modifications to the original pipeline of RBDAct: (i) Since the code was originally developed for data from GENEActiv devices [2], we converted the Axivity AX6 data into the expected format; (ii) the sleep detection approach of RBDAct relied on light sensor data; as this data was not recorded reliably by the AX6 devices, likely due to the sensor being covered by the armband, we used the sleep periods detected by the preprocessing pipeline of ActiTect. We adopted the automatically detected sleep windows instead of the sleep diaries because the latter were available for only a subset of nights, and on the 359 nights with diaries, the automated windows significantly improved iRBDAct's balanced accuracy ($p = 7.2 \times 10^{-8}$, $\delta = -0.44$), while no significant difference was observed for RBDAct ($p = 0.63$, $\delta = 0.04$). To ensure consistency with the original RBDAct implementation, we applied z-score normalization using statistics computed on the original training data. We also tested prediction using raw unscaled features, but scaling consistently improved performance. A summary of the validation of the original RBDAct model and the retrained iRBDAct model is displayed in table S1.

The original pre-trained RBDAct model failed to generalize to the iRBD cases from the CogTrAiL-RBD and Local Test datasets, with balanced accuracy near chance level, indicating substantial limitations in cross-cohort generalizability. Slightly higher AUROC and F_1 scores compared to balanced accuracy are the result of the model overpredicting the

Table S1 Results of external RBDAct validation. Classification performance of the original pretrained RBDAct and the iRBDAct retrained on CogTrAiL-RBD iRBDs, across cohorts with distinct clinical profiles: isolated RBD *(iRBD)* and Parkinson's disease with RBD *(PD+RBD)*. All metrics are expressed as mean values with 95% confidence intervals, calculated across the 100 models produced by the cross-validation scheme described in the original publication. The *Records* column shows the number of individuals (and total nights) per cohort.

Model	Cohort	Records	AUROC	F_1	Bal. Acc.
**RBDAct**	**CogTrAiL-RBD (iRBD)**	iRBD: 55 (370)<br>HC: 23 (154)	$0.62^{+0.03}_{-0.03}$	$0.62^{+0.04}_{-0.04}$	$0.53^{+0.02}_{-0.02}$
	**Local Test (iRBD)**	iRBD: 19 (119)<br>HC: 12 (79)	$0.66^{+0.01}_{-0.01}$	$0.58^{+0.04}_{-0.04}$	$0.56^{+0.01}_{-0.01}$
	**OPDC (iRBD)**	iRBD: 183 (1157)<br>HC: 60 (367)	$0.71^{+0.02}_{-0.02}$	$\mathbf{0.71^{+0.04}_{-0.04}}$	$0.57^{+0.02}_{-0.02}$
	**OPDC (PD+RBD)**	PD+RBD: 20 (128)<br>HC: 60 (367)	$\mathbf{0.81^{+0.02}_{-0.02}}$	$0.49^{+0.02}_{-0.02}$	$\mathbf{0.62^{+0.02}_{-0.02}}$
**iRBDAct**	**CogTrAiL-RBD (iRBD)**	iRBD: 55 (370)<br>HC: 23 (154)	$\mathbf{0.83^{+0.02}_{-0.02}}$	$0.79^{+0.01}_{-0.01}$	$\mathbf{0.77^{+0.01}_{-0.01}}$
	**Local Test (iRBD)**	iRBD: 19 (119)<br>HC: 12 (79)	$0.65^{+0.01}_{-0.01}$	$0.65^{+0.01}_{-0.01}$	$0.62^{+0.01}_{-0.01}$
	**OPDC (iRBD)**	iRBD: 183 (1157)<br>HC: 60 (367)	$0.78^{+0.02}_{-0.02}$	$\mathbf{0.81^{+0.02}_{-0.02}}$	$0.72^{+0.01}_{-0.01}$
	**OPDC (PD+RBD)**	PD+RBD: 20 (128)<br>HC: 60 (367)	$0.80^{+0.02}_{-0.02}$	$0.58^{+0.02}_{-0.02}$	$0.73^{+0.02}_{-0.02}$

Bold: Indicates the highest value of each metric across all cohorts.

RBD class in combination with a class imbalance. When evaluated on the OPDC (iRBD) cohort, all metrics improve significantly $\left(p \leq 1.4 \times 10^{-4}\right)$ compared to the CogTrAiL-RBD and Local Test cohorts, except balanced accuracy ($p = 1.6 \times 10^{-3}$, $\delta = -0.26$ vs. Cologne train; $p = 0.80$, $\delta = 0.02$ vs. Cologne test), indicating that gains in AUROC and F_1 do not reflect a balanced improvement in sensitivity and specificity. Within the OPDC (PD+RBD) cohort, RBDAct demonstrates a more balanced classification performance with a significant increase $\left(p \leq 1.5 \times 10^{-3}\right)$ in AUROC and balanced accuracy compared to the iRBD cohorts. The lower F_1 score likely reflects a reduction in recall due to fewer false positives, consistent with more conservative predictions. A potential explanation is that RBDAct was originally trained on a PD+RBD cohort and evaluated here on iRBD cases. However, while there is no definitive clinical consensus, current evidence tends to suggest no substantial differences in motor event characteristics between PD+RBD and iRBD [3, 4].

When retrained on iRBD cases from the CogTrAiL-RBD cohort, the iRBDAct model shows significantly $\left(p \leq 2.8 \times 10^{-11}, \delta \leq -0.55\right)$ improved balanced accuracy across all cohorts, including the OPDC (PD+RBD) cohort. This suggests that differences in motor signatures between PD+RBD and iRBD may be less impactful on model performance than the benefits of increased training data size. The iRBDAct model, retrained on the CogTrAiL-RBD set, performs significantly better under cross-validation than on the test data across all evaluation metrics ($p \leq 1.2 \times 10^{-19}$, $\delta \leq -0.74$), indicating a drop in performance when generalizing beyond the training distribution.

Taken together, these results indicate that the original pretrained RBDAct models failed to generalize to two external and independent cohorts, with balanced accuracy approaching chance level even on PD+RBD cases. While increasing training data size and retraining on iRBD cases improved classification performance across all scenarios, generalization remained limited. Combined with practical constraints—such as device incompatibility

and reliance on light-sensor-based sleep detection—these findings underscore the need for a more robust and generalizable actigraphy-based screening pipeline.

Supplementary B List of Engineered RBD Motor Pattern Features

Table S2 **Overview of Engineered Motion Features**

Level	Group	Numerical Features	Axes	Interpretation
**Local**	**Distributional**	mean, std, skew, kurt, quantile($q = 0, 0.25, 0.5, 0.75, 1.0$)	$a_{x,y,z}$, $\|\|\vec{a}\|\|_2$	Strength, range, dispersion and skewness of movement amplitude.
	**Energy**	SMA, Power, RMS	$a_{x,y,z}$, $\|\|\vec{a}\|\|_2$	Integrated movement intensity.
	**Spectral**	$\{f_1, f_2, f_3\} = \arg\max_f \mathrm{ASD}(f)$, $\mathrm{ASD}\,\|_{f \in \{1,2,4,8,16,f_1,f_2,f_3\}\ \mathrm{Hz}}$, $\sum_f$ ASD, entropy(ASD)	$\|\|\vec{a}\|\|_2$	Separation between slow & coordinated movements or rapid & jerky motor actions.
	**Auto-Correlation**	$\mathrm{AC}(\tau)\|_{\min}^{\max}, \tau\|_{\min}^{\max}, \mathrm{N}_{\mathrm{zero}}(AC)$	$a_{x,y,z}$, $\|\|\vec{a}\|\|_2$	Periodicity and rhythmicity of motions.
	**Peaks**	$\mathrm{N}_{\mathrm{peaks}}$ /sec, avg(Prom),min/max(Prom)	$\|\|\vec{a}\|\|_2$	Fragmentation of movement.
	**Non-linear Dynamics**	SampEn, $\mathrm{Hurst}_{\mathrm{rs}}$	$a_{x,y,z}$, $\|\|\vec{a}\|\|_2$	Predictability, complexity, and memory of movement.
	**Poincaré**	SD_1, SD_2, $\mathrm{A}_{\mathrm{ellipse}}$	$\|\|\vec{a}\|\|_2$	Long and short term variability.
	**Duration**	Δt	$\|\|\vec{a}\|\|_2$	Duration of movement bout.
**Global**	**Clusters**	Hopkins H, $\mathrm{KDE}_{\mathrm{moves}} \rightarrow$ peaks + prom	$\|\|\vec{a}\|\|_2$	Degree of clustering or dispersion of movements throughout the night.
	**Number of Movements**	$\mathrm{N}_{\mathrm{moves}}$ /h	$\|\|\vec{a}\|\|_2$	Overall activity during the night.
	**Inter-Event Intervals**	$\mathrm{IEI}_{\mathrm{moves}}$	$\|\|\vec{a}\|\|_2$	Movement spacing and irregularity across the night.

Supplementary C Hyperparameter Search Space

Table S3 Hyperparameter Search Space for XGBoost

Name	Interpretation	Range	Type
n_estimators	Number of boosting trees	[300, 1100]	Integer (uniform)
max_depth	Maximum individual tree depth	[6, 12]	Integer (uniform)
min_child_weight	Minimum sum Hessian (min child weight)	[1, 15]	Real (log-uniform)
learning_rate	Shrinkage step size	[0.02, 0.12]	Real (log-uniform)
subsample	Row subsampling ratio	[0.55, 0.95]	Real (uniform)
colsample_bytree	Feature subsampling per tree	0.5	Fixed to default
colsample_bylevel	Feature subsampling per tree level	0.3	Fixed to default
reg_alpha	L1 regularization strength	0.0	Fixed to default
reg_lambda	L2 regularization strength	1.0	Fixed to default
top_k_feats	Number of selected features (custom)	[5, 35]	Integer (uniform)

Supplementary D Per Dataset Ranking Correlation

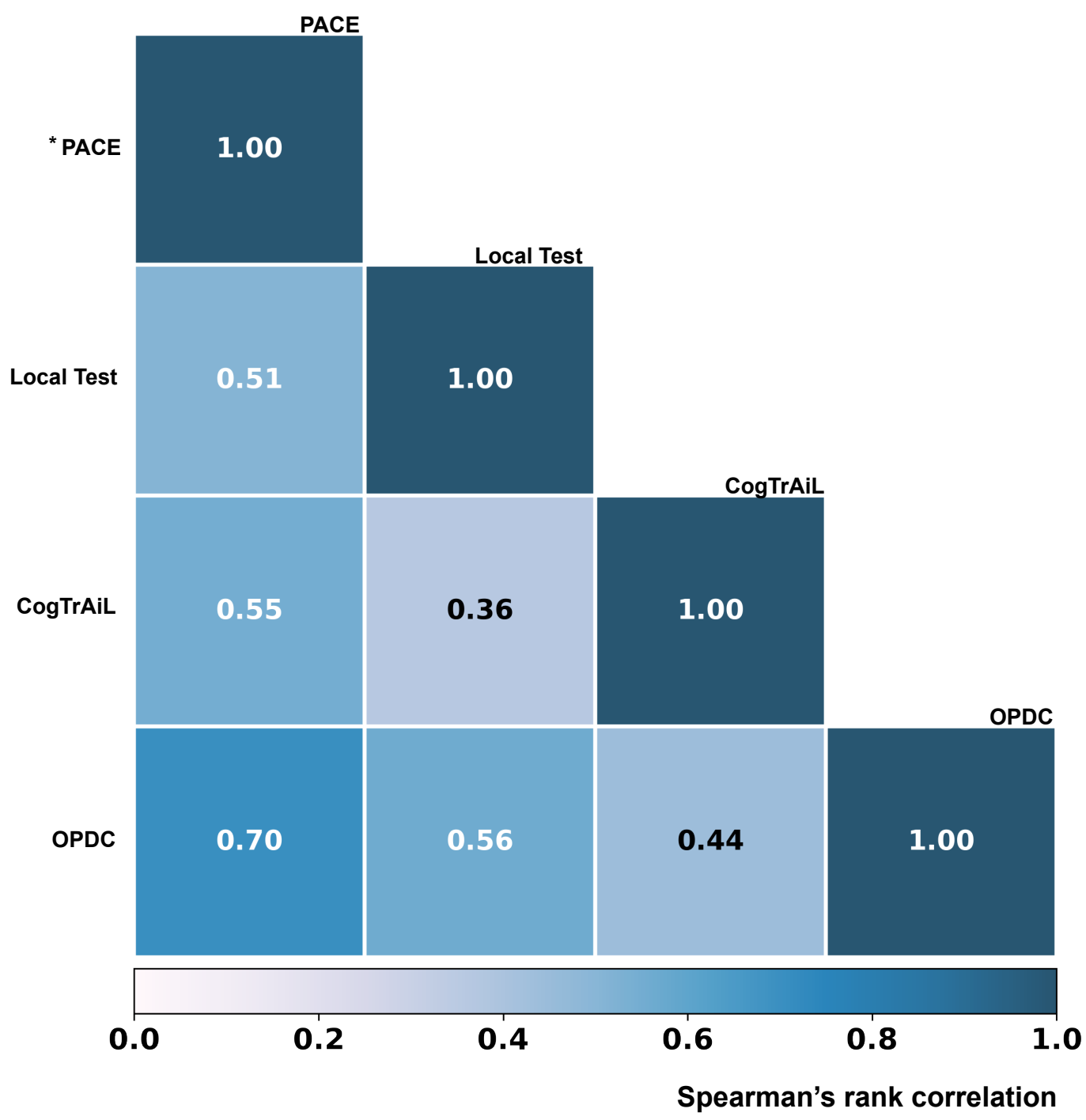

Figure S1 Spearman's rank correlations of feature importance rankings derived within individual datasets. Correlations ranged from 0.36 to 0.70, indicating moderate-to-strong agreement overall while still reflecting cohort-specific feature preferences. This variability underscores the value of pooling data to optimize the generalizability of feature sets.

Supplementary E Multi-Center Model Validation (including Local Test)

Table S4 Multi-Center Model Validation (including Local Test). Displayed are classification metrics across LODO folds when Local Test is part of the evaluation. The confidence intervals (95%) are estimated via stratified bootstrap ($n = 2000$). Upper block: all-RBD; lower block: iRBD-only.

Cohort	Records		AUROC	F_1	Bal. Acc.
**PACE (all-RBD)**	iRBD:	23 (171)	$0.841^{+0.004}_{-0.004}$	$0.881^{+0.002}_{-0.002}$	$0.702^{+0.004}_{-0.004}$
	PD+RBD:	19 (117)			
	PD–RBD:	9 (38)			
	HC:	6 (57)			
**Local Test (all-RBD)**	iRBD:	19 (119)	$0.813^{+0.004}_{-0.004}$	$0.703^{+0.004}_{-0.004}$	$0.721^{+0.004}_{-0.004}$
	HC:	12 (79)			
**CogTrAiL (all-RBD)**	iRBD:	19 (119)	$0.895^{+0.002}_{-0.002}$	$0.812^{+0.002}_{-0.002}$	$0.810^{+0.002}_{-0.002}$
	HC:	12 (79)			
**OPDC (all-RBD)**	iRBD:	80 (496)	$0.782^{+0.003}_{-0.003}$	$0.903^{+0.001}_{-0.001}$	$0.679^{+0.002}_{-0.002}$
	PD+RBD:	8 (50)			
	HC:	25 (144)			
**PACE (iRBD)**	iRBD:	23 (171)	$0.974^{+0.002}_{-0.002}$	$0.957^{+0.002}_{-0.002}$	$0.961^{+0.002}_{-0.002}$
	HC:	6 (57)			
**Local Test (iRBD)**	iRBD:	19 (119)	$0.747^{+0.004}_{-0.004}$	$0.659^{+0.004}_{-0.004}$	$0.636^{+0.004}_{-0.004}$
	HC:	12 (79)			
**CogTrAiL (iRBD)**	iRBD:	19 (119)	$0.882^{+0.002}_{-0.002}$	$0.790^{+0.002}_{-0.002}$	$0.778^{+0.002}_{-0.002}$
	HC:	12 (79)			
**OPDC (iRBD)**	iRBD:	80 (496)	$0.806^{+0.003}_{-0.003}$	$0.908^{+0.001}_{-0.001}$	$0.691^{+0.002}_{-0.002}$
	HC:	25 (144)			

Supplementary F Statistical Significances of Presented Features Per Dataset

Table S5 Statistical Significances of Presented Features Per Dataset. P-values are computed using one-sided Mann-Whitney-U test and are presented alongside effect sizes, expressed as Cliff's Delta. The sample sizes (N) per dataset are given as RBD/HC. Statistically signifcant values (<0.05) are marked bold.

		**Pooled**	**CogTrAiL**	**Local Test**	**OPDC**	**PACE**
		N=1334/499	N=370/154	N=119/79	N=546/144	N=299/122
**Power Skewness**	p-value	$\mathbf{4.5 \times 10^{-42}}$	$\mathbf{3.6 \times 10^{-12}}$	**0.002**	$\mathbf{3.1 \times 10^{-19}}$	$\mathbf{2.2 \times 10^{-9}}$
	Cliff's δ	-0.41	-0.39	-0.26	-0.49	-0.37
**First Autocorr. Minimum**	p-value	$\mathbf{5.7 \times 10^{-17}}$	$\mathbf{6.4 \times 10^{-13}}$	**0.001**	$\mathbf{3.1 \times 10^{-8}}$	$\mathbf{7.8 \times 10^{-10}}$
	Cliff's δ	0.25	0.40	0.28	0.30	0.38
**Peak Frequency IQR**	p-value	$\mathbf{2.9 \times 10^{-7}}$	$\mathbf{1.0 \times 10^{-12}}$	$\mathbf{4.2 \times 10^{-5}}$	0.660	**0.019**
	Cliff's δ	-0.16	-0.40	-0.34	-0.02	-0.15
**Median Bout Duration**	p-value	$\mathbf{4.6 \times 10^{-94}}$	$\mathbf{3.0 \times 10^{-32}}$	$\mathbf{9.1 \times 10^{-12}}$	$\mathbf{7.9 \times 10^{-24}}$	$\mathbf{4.0 \times 10^{-28}}$
	Cliff's δ	0.62	0.66	0.57	0.54	0.68
**Spectral Entropy SD**	p-value	$\mathbf{2.4 \times 10^{-50}}$	$\mathbf{2.7 \times 10^{-12}}$	$\mathbf{7.2 \times 10^{-7}}$	$\mathbf{2.8 \times 10^{-18}}$	$\mathbf{7.2 \times 10^{-16}}$
	Cliff's δ	-0.45	-0.39	-0.42	-0.47	-0.50

Supplementary G Medication Prevalence Per Dataset

Medication metadata were collected across cohorts at the closest clinical encounter to actigraphy, not always contemporaneously with the actigraphy nights themselves, which is a limitation but reflects real-world recording conditions.
We grouped medication into potentially influential (**bold**) for (sleep) motor movements versus non-influential drugs (non-bold) based on prior literature demonstrating effects on REM sleep physiology, RSWA or (nocturnal) motor behavior in general [5–13]:

- **Melatonin**: can alter REM physiology and is recommended for symptomatic RBD treatment. [5, 6]
- **Clonazepam / Benzodiazepines**: Sedative/motor-suppressing; commonly used for RBD. [5, 6]
- **Z-drugs**: Z-drugs alter sleep architecture (sleep stage timing, amount of REM, etc.) and have been associated with complex sleep-related behaviours, although no consistent direct effects on REM motor atonia have been demonstrated. [9] Examples: Zopiclone, Zolpidem, Doxylamine etc.
- **Antidepressants**: Associated with increased RSWA / RBD-like features in some individuals. [7, 8] Examples: Citalopram, Sertraline, Mirtazapine, etc.
- **Mood Stabilizers / Antiseizure Meds**: Can have an influence on the central-nervous system (CNS); may cause tremor/sedation. [10, 11] Examples: Sodium Valproate, Lamotrigine, Lithium etc.
- **Other Sleep- or CNS-active Meds**: Includes medications with central nervous system effects that can influence sleep architecture, arousal, or psychomotor function. Gabapentinoids such as pregabalin have been shown to alter sleep motor correlates and induce somnolence/ataxia, while CNS stimulants like methylphenidate and modafinil modulate dopaminergic/noradrenergic systems relevant to motor control and wakefulness. [12] Examples: Gabapentin, Pregabalin, Methylphenidate, Modafinil, etc.
- Supplements / Alternative Medicine: General, mostly non-prescription supplememts or medication. Such are mostly studied for nutritional and sleep-quality effects, not directly on motor impact. For example, Magnesium supplementation has modest effects on sleep-quality and duration, but there's no strong evidence of direct motor system modulation at typical doses. [13] Examples: Vitamins, Magnesium, etc.
- Antiplatelet / Anticoagulants: Such medication primarily act peripherally or via metabolic pathways and do not affect the CNS. Hence, we assume no direct motor effects. Examples: Aspirin, Clopidogrel, Apixaban.
- Lipid-lowering: Assumed to a have neglible motor effects, demonstrated to have no infleunce on any cognitive domain. [14] Examples: Atorvastatin, Ezetimibe, Rosuvastatin.
- Gastrointestinal: Act peripherally on the digestive system, no evidence on direct affect of central motor control or motor patterns during sleep, if at all, via only indirect via symptoms/arousals. Examples: Omeprazole, Lansoprazole, Gaviscon.
- Other System Meds: Other, non-specific meds like Pain / Inflammation, Urological, Antidiabetics etc. Similiar to Gastrointenstinal medication expected to have none, or negligble direct impact on nocturnal motor patterns. Examples: Ibuprofen, Paracetamol, Metformin, Levothyroxine, Tamsulosin etc.

Table S6 Medication prevalence across cohorts. Values are patient-level counts per diagnosis subgroup. Medication is grouped into categories which are either considered as potentially motor-influential (**bold**) or not (non-bold).

	**CogTrAiL-RBD**		**Local Test**		**OPDC**			**PACE**			
	iRBD	HC	iRBD	HC	iRBD	PD+RBD	HC	iRBD	PD+RBD	PD-RBD	HC
**Samples**[a]	55	23	19	12	70 (80)	8 (8)	25 (25)	13 (23)	10 (19)	5 (9)	3 (6)
**Melatonin**	5	na	-	-	21	2	-	5	-	-	-
**Clonazepam / Benzodiazepines**	1	na	-	-	33	-	-	3	-	-	-
**Z-drugs**	-	na	-	1	2	-	-	-	1	-	-
**Antidepressants**	3	na	2	1	18	-	3	-	2	-	-
**Mood stabilizers / Antiseizure Meds**	2	na	-	2	1	-	-	-	-	-	-
**Other Sleep- or CNS-active Meds**	-	na	-	-	6	-	1	1	1	1	-
$\sum$ any [b]	10	na	2	2	48	2	4	9	2	1	-
Supplements / Alternative Medicine	14	na	4	2	10	-	2	na	na	na	na
Antiplatelet / Anticoagulants	12	na	5	1	17	3	3	na	na	na	na
Lipid-lowering	17	na	4	3	28	1	13	na	na	na	na
Gastrointestinal	8	na	1	1	20	1	9	na	na	na	na
Other Systemic Meds	38	na	12	7	na	na	na	na	na	na	na

na: Data not available.
[a] **Number of contributed recordings**: For CogTrAiL-RBD and Local Test, this is equal to the number of unique subjects and actigraphy recordings. For PACE and OPDC, its equal to N_{subjects} (N_{records}), as some subjects contributed multiple recordings. For PACE, only recordings with ≥ 4 nights are shown.
[b] **Number of subjects with any motor-influential medication**: Count of subjects with medication from any of the as potentially motor-influential classified categories, some subjects have medication in multiple categories.

Across cohorts, potentially motor-influential medication had higher prevalence within RBD-positive subgroups. When considering *any* motor-influential medication, prevalence varied across cohorts. In OPDC, 50 of 70 RBD positive subjects (iRBD & PD+RBD) and 2 of 25 HC subjects had at least one motor-influential medication recorded. In PACE, 11 of 23 RBD positive and 1 of 8 RBD negative (HC & PD–RBD) had at least one such medication. In Local Test, motor-influential medication was observed in both RBD (2/19) and HC (2/12) participants. CogTrAiL-RBD only recorded motor-influential medication for iRBD subjects (10/55), with no HC medication data available.

References

[1] Raschellà, F., Scafa, S., Puiatti, A., Martin Moraud, E. & Ratti, P.-L. Actigraphy Enables Home Screening of Rapid Eye Movement Behavior Disorder in Parkinson's Disease. *Annals of Neurology* **93**, 317–329 (2023).

[2] Activinsights Ltd., GENEActiv Product Information Sheet (accessed 15.04.2025). URL https://activinsights.com/wp-content/uploads/2022/06/GENEActiv-Product-Information-Sheet.pdf.

[3] Bugalho, P. *et al.* Characterization of motor events in REM sleep behavior disorder. *Journal of Neural Transmission* **124** (2017).

[4] Assogna, F. *et al.* Cognitive and Neuropsychiatric Profiles in Idiopathic Rapid Eye Movement Sleep Behavior Disorder and Parkinson's Disease. *Journal of Personalized Medicine* **11**, 51 (2021).

[5] Standards of Practice Committee *et al.* Best Practice Guide for the Treatment of REM Sleep Behavior Disorder (RBD). *Journal of Clinical Sleep Medicine* **6**, 85–95 (2010).

[6] McCarter, S. J. *et al.* Treatment outcomes in REM sleep behavior disorder. *Sleep Medicine* **14**, 237–242 (2013).

[7] Postuma, R. B. *et al.* Antidepressants and REM Sleep Behavior Disorder: Isolated Side Effect or Neurodegenerative Signal? *Sleep* **36**, 1579–1585 (2013).

[8] McCarter, S. J. *et al.* Antidepressants Increase REM Sleep Muscle Tone in Patients with and without REM Sleep Behavior Disorder. *Sleep* **38**, 907–917 (2015).

[9] Louzada, L. L., Machado, F. V., Nóbrega, O. T. & Camargos, E. F. Zopiclone to treat insomnia in older adults: A systematic review. *European Neuropsychopharmacology* **50**, 75–92 (2021).

[10] Zhou, D. J. *et al.* Movement disorders associated with antiseizure medications: A systematic review. *Epilepsy & Behavior* **131**, 108693 (2022).

[11] Dols, A. *et al.* The prevalence and management of side effects of lithium and anticonvulsants as mood stabilizers in bipolar disorder from a clinical perspective: a review. *International Clinical Psychopharmacology* **28**, 287 (2013).

[12] Kallweit, M. S., Kallweit, N. P. & Kallweit, U. Pharmacological Treatments of Sleep–Wake Disorders: Update 2023. *Clinical and Translational Neuroscience* **7**, 42 (2023).

[13] Zhang, Y. *et al.* Association of magnesium intake with sleep duration and sleep quality: findings from the CARDIA study. *Sleep* **45**, zsab276 (2022).

[14] Li, K., Li, Y., Jiang, X. & Zhu, Y. The effects of cholesterol-lowering drugs on neurocognitive function: systematic review and meta analysis. *Frontiers in Neurology* **17** (2026).
